# A WICS-based model for women’s leadership

**DOI:** 10.3389/fpsyg.2025.1586457

**Published:** 2025-08-11

**Authors:** Christiana Papadakou, Robert J. Sternberg

**Affiliations:** ^1^Department of Applied Psychology, Steinhardt School of Culture, Education, and Human Development, New York University, New York, NY, United States; ^2^Department of Psychology, College of Human Ecology, Cornell University, Ithaca, NY, United States

**Keywords:** leadership, gender and leadership, women leadership, leadership model, leadership trait analysis, wisdom-intelligence-creativity-synthesized, WICS

## Abstract

Men have typically dominated in leadership positions for what we have of recorded history; hence, leadership has been a concept whose study has revolved mostly around men. Nowadays, however, more and more women are emerging in the political field (as well as in other fields), taking up significant political and other roles, including leading political parties and countries. Women and men often differ in their leadership styles. Both men and women have, on average, variegated patterns of strengths and weaknesses. This article examines characteristics of successful women leaders. The article discusses and analyzes these characteristics, using as a theoretical framework the Wisdom-Intelligence-Creativity-Synthesized (WICS) model of leadership. Present and recent leaders like Jacinda Ardern, Sanna Marin, Ursula von der Leyen, and more, will be discussed in relation to the proposed characteristics. The proposed characteristics include, among others, the three C’s of consideration, caring, and compassion; humility; unity; diplomacy; and resilience to the misogynistic critiques they often receive. Of course, it is of great significance to mention that no leader is perfect, and like all leaders, the aforementioned ones did some things in their leadership positions that worked and other things that did not.

## Introduction

In the last century, a number of notable women leaders have emerged in the political field as well as in other fields (Alan et al., 2020; Burke and Collins, 2001; De la Rey, 2005). Many of them have proven to be highly competent leaders of political parties and even countries (Bass et al., 1996; Blake-Beard et al., 2020; Craig, 2021; Gipson et al., 2017). Through their competencies, they have helped to bridge the gap between perceptions of the leadership skills of men and women. They also have helped to amend misconceptions surrounding women’s competencies, leadership, and management skills, as well as claims to equity in leadership positions. Because men have predominantly been in power most of the time and in most places in recorded history, leadership, in most sectors of society, has been largely a male entitlement and privilege (Eagly and Karau, 2002; Koenig et al., 2011); hence, leadership has been a concept whose study has revolved around men (De Nmark, 1993).

Most existing models of leadership, therefore, are still very much focused around the stereotypical characteristics of men leaders (De Nmark, 1993; Eagly and Carli, 2007; Ryan and Dickson, 2018). As a result, if applied to women, these models may fail to adequately recognize and showcase their strong leadership qualities and skills, and would highlight what would appear to be weaknesses. There seems to be, therefore, a gap in the literature: a fair and holistic model to characterize successful women’s leadership. This manuscript serves as an attempt to bridge the gap in the leadership literature, and to suggest and provide the development of a model of successful women’s leadership. Such a model will acknowledge and value the particular skills and competencies of outstanding women leaders. Such a model also will recognize possible limitations that can derive from the proposed characteristics.

## Existing literature on women leadership

Leadership is still considered a predominantly masculine domain, especially by people who hold traditional gender-role and leadership-role stereotypes (Eagly and Carli, 2007). Role Congruity Theory posits that a group will be positively evaluated when its expressed characteristics are thought to be aligned with that group’s typical social roles; according to this theory, prejudice against women leaders often derives from the degree of incongruence between stereotypes and social norms and stereotypes about leadership (Eagly and Diekman, 2005; Eagly and Karau, 2002). Essentially, when a woman leader demonstrates leadership traits and exercises leadership practices, an incongruence is created between the perceived female role and the perceived leadership role, which makes the perceiver hold a somewhat less favorable perception of the woman leader (Heilman and Okimoto, 2007; Heilman et al., 2004; Wang et al., 2013). Another theory that describes largely the same concept, that of leadership being viewed through a masculine lens as a default, has been proposed by Ryan and Dickson (2018). Ryan and Dickson (2018) referred to an ‘invisible norm,’ and argued that the issue in gender and leadership is not an underrepresentation of women, but rather “the dominant presence of groups of men and valued forms of masculinities.”

A notable common difference between women and men in terms of leadership style is the transformational versus transactional style (Bass et al., 1996; Goethals and Hoyt, 2016). Transactional leadership refers to a more mundane exchange between leaders and followers, in which each party does something for the other (Odumeru and Ogbonna, 2013)—a tit for tat mentality of leadership and followership. Transformational leadership refers to a more engaging form of leadership, in which the leader and the followers work collaboratively to achieve results that create positive change, and which are beyond the leader’s immediate self-interest (Bass et al., 1996). A transformational leader inspires and motivates their followers to perform beyond their perceived capabilities. Especially relevant are the findings by Eagly et al. (2003) on their meta-analysis of 45 studies of transformational, transactional, and laissez-faire leadership styles. Their findings revealed that women leaders were overall more likely than men leaders to adopt transformational leadership approaches–which are positively associated with leadership effectiveness. In contrast, men leaders were more likely to exhibit transactional leadership styles, including active and passive management by exception, which tends to correlate with lower effectiveness. These findings align with the consensus in the literature that men are viewed by many as more likely to demonstrate leadership characteristics that align with a transactional style of leadership (Bass et al., 1996; Burke and Collins, 2001; Goethals and Hoyt, 2016; Guillet et al., 2019), and women are viewed by many as more likely to demonstrate leadership characteristics that align with a transformational style of leadership (Eagly and Carli, 2003; Eagly et al., 2003).

In the literature on leadership, it would appear that most existing models of leadership (see Charan et al., 2011; Hermann, 2005; Organ, 1996) have been developed based largely on the predominant men’s leadership styles, without taking into consideration the different and especially effective leadership styles that women often bring to the table. As a result, the focus of models of leadership is on leadership characteristics that are mostly considered ‘masculine.’ If applied to women, these models may fail to adequately showcase the strong leadership qualities and skills of women. Indeed, the chances are that the opposite might happen–these models might highlight what would appear to be weaknesses among women leaders. However, if these models were applied to men leaders, it is likely that the models would successfully highlight the men’s strong leadership qualities and skills. Therefore, one could say that there is an underlying degree of gender disparity in the way leadership models are built.

### Are existing models more men-focused than women-focused?

An example primarily portraying this masculine perspective on leadership is the model of Leadership Trait Analysis (Hermann, 2005). This model consists of seven characteristics: belief in ability to control events, need for power, conceptual complexity, self-confidence, task orientation, in-group bias, and distrust of others. This model, on average, serves men leaders better than women leaders, due to the stereotypically masculine traits of which it consists, which will be analyzed further below. Reviewing some of the traits it focuses on, one can assess whether those traits would benefit both men and women leaders equally in the evaluation of leadership.

Women tend to report lower self-confidence than men. This difference has been demonstrated in multiple studies across diverse environments, showing consistency across professional, educational, and interpersonal settings. Women express more self-doubt (Kay and Shipman, 2014), negotiate less and ask for lower salaries (Small et al., 2007), and underestimate their abilities despite equal performance (Ehrlinger and Dunning, 2003). The need for power can be defined as the internal motivation and need to exercise influence and control over others, and to be recognized and encouraged (Bennett, 1988). Men, on average, value male-typical attributes more than women do, including attributes such as earnings, power, and promotions (Konrad et al., 2000).

As a general notion, men seem to attribute more significance in leadership to prestige, respect, awe, and power than women do (O’Connor, 2001). Additionally, there is a stereotypical notion that task-oriented leadership is more masculine; on the other hand, a relationship-oriented approach is considered more feminine (Cann and Siegfried, 1990; Ridgeway, 2001). Men, especially of high status, have also proven, on average, to have a stronger in-group bias than do women, expressing evaluative bias towards said group (Scheepers et al., 2006). From the brief inspection of the characteristics Hermann’s Leadership Trait Analysis focuses on (self-confidence, need for power, task-orientation, and in-group bias), it appears that this particular model is primarily tailored toward men, potentially favoring them when used to assess leadership. It may not necessarily well characterize the attributes of women leaders.

Hermann’s (2005) Leadership Trait Analysis model is not the only model that seems to be built around characteristics of successful men leaders. When considering theories of leadership, either from their content, or simply their names, it seems as though there are quite a few leadership models that are male-centered.

A popular theory of leadership is the Leadership Pipeline model (Charan et al., 2011), which provides a framework that one can utilize to identify future leaders and plan their leadership development. It consists of six steps, such as “managing self to managing others” (step 1), “managing others to leading managers” (step 2), and “group manager to enterprise manager” (step 6). This pipeline of management and leadership, although rational and efficient, could also serve as an illustration of leadership models that exhibit gender bias, for two reasons.

First, although there have been advancements and changes in gender equality and opportunities for women in leadership over the past couple decades, the majority of leadership, managerial, and CEO positions, still tend to be occupied by men (Eagly and Karau, 2002; Koenig et al., 2011; McDonagh et al., 2014; Oakley, 2000; Steffens et al., 2019). Second, men often receive more promotions than women, and women frequently face stricter criteria for promotions and advancement (Ibarra et al., 2010; Lyness and Heilman, 2006). Therefore, in a theory in which the first step is becoming a manager and the latter steps are about advancing through higher managerial roles, gender equality can be hindered by the uneven distribution of men versus women in managerial positions and in pipelines for promotions. Although this theory may revolve primarily around leadership in the context of business, it still adds to the larger idea that leadership is predominantly a masculine concept.

Last, there is the Great Man Theory of Leadership (Organ, 1996), which posits the inborn and fixed nature of leadership traits, as well as the notion of how people in positions of power come to lead because of traits granted to them at birth. These traits, in principle, might seem unrelated to gender; still, it cannot be overlooked that the name of the theory clearly demonstrates that this leadership theory automatically characterizes men, not women. When this theory was created, the idea of a serious and successful woman leader was something practically unheard of (Moran, 1992).

### Leadership styles: variation within sex

Women and men typically differ somewhat in their leadership styles (Burke and Collins, 2001). This difference does not signify that one style is necessarily better than the other, nor that women and men in leadership need to be contrasted with each other with the intention of putting one above the other. Both typical women and men leadership styles have potential strengths and weaknesses. In addition, there is no single women or men leadership style; there is large variation within sex, and there are multiple examples of this. Former UK Prime Minister Margaret Thatcher was known for her strong and decisive leadership style, earning her the nickname “The Iron Lady” (Dyson, 2009), whereas former Chancellor Angela Merkel of Germany was well-known for her pragmatic, safe, and steady approach to leadership, particularly when working to navigate, not always successfully, the Eurozone financial crisis and refugee crisis (Mayer and Niekerk, 2020; Yoder, 2011).

Indira Gandhi, India’s first woman Prime Minister, was an ambitious and dominating political leader known for her astute and assertive leadership during a period of significant change (Steinberg, 2005). Her leadership style has often been regarded as dogmatic and even masculine. So has the leadership style of Israel’s fourth Prime Minister, Golda Meir (Banai and Mayer, 2023). On the other hand, New Zealand’s recent Prime Minister, Jacinda Ardern, was recognized for her empathetic and compassionate leadership style, especially during crises such as the Christchurch Mosque shootings and the COVID-19 pandemic (Brown and Schockman, 2022; Johnson and Williams, 2020). This is not to say that any of these women leaders was perfect–no leader is. However, they achieved success by demonstrating strengths in leadership qualities commonly associated with effective women leaders (Eagly and Carli, 2003).

In the realm of men leadership, Winston Churchill, known for his strong and inspirational leadership during World War II as the Prime Minister of the United Kingdom, adopted a leadership style that was resolute and charismatic (Best, 2001; Rao, 2010). Meanwhile, Franklin D. Roosevelt, as the President of the United States, skillfully guided the nation through the Great Depression and World War II with a calm and reassuring demeanor (Stuckey, 2013). South Africa’s first black President, Nelson Mandela, was celebrated for his leadership characterized by reconciliation and a commitment to peacefully ending apartheid; his style has often been labeled as servant leadership (Falzon, 2015), also known as “leading from behind.” These examples display the coexistence of various leadership styles both among and within genders, demonstrating that no single style can claim dominance or superiority.

### Why is a is a model focusing on women’s leadership style needed?

The purpose of leadership models is to enhance our comprehension of leadership, classify and clarify dimensions of active leadership, and provide a framework for assessing and evaluating leaders’ performance (Nawaz and Khan, 2016). Leadership models generally are not intentionally biased toward any gender. Consequently, proposing new models should not be viewed as an attempt to favor one gender over the other. However, given that some existing models may inadvertently favor characteristics associated with men leaders, it is a matter of equity to develop analogous models that highlight the unique strengths and qualities of many successful women leaders, without diminishing the observed effectiveness of men leaders. This approach ensures a more balanced and inclusive perspective on leadership.

To proceed with the theoretical framework of a proposed model, one needs to acknowledge how stereotypes, prejudice, and discrimination can serve as a foundation to explain perceived effectiveness in leadership styles (Gipson et al., 2017). Models like the ones examined above can contribute to the mistaken belief that, for a leader to be successful, they need to possess predominantly masculine traits. This belief strengthens prejudice against women leaders. Prejudices flourish through stereotypes about social groups that are contrary to the traits that are considered necessary for success in leadership roles (Eagly and Karau, 2002).

There are numerous differences in the expectations for the behavior of men versus women in a workplace environment or similar settings, due to perceived descriptive and prescriptive stereotypes (Eagly and Karau, 2002; Heilman, 1983). Prejudice toward women leaders stems in part from stereotypical perceptions of women being in contrast with the stereotypical perceptions of successful leaders, which tend to be masculine (Eagly and Karau, 2002). As a result of the contrasting nature of these two, an incompatibility is often created upon exposure to the leadership of a woman leader. There is an abundance of empirical evidence demonstrating that people consider successful leaders to be closer to their perception of an ideal man than of an ideal woman (Heilman et al., 1989; Lee and Hoon, 1993; Rosenwasser and Dean, 1989; Schein, 1973; Schein, 1975).

This difference is also demonstrated in the negative commentary that women leaders sometimes receive; oftentimes, negative feedback on women leaders will be based on a stereotypical idea of femininity (e.g., emotional, irrational), portraying the woman leader as lacking competence (Sakki and Martikainen, 2021). An example portraying this exaggerated attention to feminine stereotypes, as an offense and proof of incompetence, can be found in the backlash former Finland Prime Minister Sanna Marin received after doing a photoshoot for *Trendi* magazine, in October 2020. The photoshoot was considered by some to be provocative and inappropriate for a Prime Minister (Sakki and Martikainen, 2021). Marin was wearing a suit that was left unbuttoned, clearly without wearing a shirt below the suit, which left her cleavage exposed. The feedback she received was mocking, hateful, and negative; many of the comments focused on questioning Marin’s intelligence and portraying her as irrational and incompetent. The feedback referred to the perceived inappropriateness of the photoshoot as proof of the Prime Minister’s stupidity and inability in her position (Andi and Selva, 2020; Sakki and Martikainen, 2021).

In a similar manner, men leaders have been photographed in more provocative ways as well. An example is demonstrated by current President of Russia Vladimir Putin, who has appeared more than once shirtless, while undertaking various activities in nature, such as riding horses, holding rifles, or swimming. His shirtless holiday pictures are typically released by the Kremlin, which is the seat of the Russian government (Foxall, 2013). Scholars and newsletter platforms have reacted in a way quite different than to Marin’s provocative photos. Foxall (2013), in a paper published in *Geopolitics*, characterized Putin’s nudity as a “cultural product that connects the viewer, through the body of Putin, to the scale of the nation.” Foxall (2013) further added that this body-nation connection is “necessary for the construction of a territorially bounded state occupied by a cohesive nation,” and that the pictures capture Putin’s “domination of space, […] technology, nature, [and] his own emotions, and other bodies.” Putin has also been compared to James Bond and Jack Ryan, since the emergence of such pictures–both fictional characters often portrayed as embodiments of power, competence, and physical attractiveness (Foxall, 2013).

In an article titled “How bizarre, ultra-manly photos help Putin stay in power,” Taub and Harris (2015) discussed how Putin’s rifle pictures make him seem powerful, and highlight the greatness of Russia, including the country’s natural resources and rich wildlife. It has also been mentioned how these pictures provide an underlying reminder to the Russian public: that their leader is healthy, strong, and in control (Taub and Harris, 2015). Putin himself, when asked by Austrian reporter Armin Wolf about these pictures, responded by saying “When I am on vacation, I see no need to hide behind the bushes, and there is nothing wrong with that,” in addition to him pointing out that he is pictured half-naked, and not naked (Dharni, 2021).

Given the political events involving Kamala Harris running in 2024 against Donald Trump for the United States presidency, it is worth shedding light on the treatment of these two figures and investigating whether they were held to different standards, potentially due to their sex. As Väyrynen (2024) wrote, after collecting data from 67 online news articles, the representations of these two political figures were divided into two main categories: political and personal representations. A majority of the political representations were related to Trump, but a majority of the personal representations had to do with Harris. Harris’ gender and race were discussed extensively, and criticized disproportionately, alongside various misogynist and sexist comments about her. Harris was described as unable to fix the American economy, and Trump was discussed as someone whose knowledge of economics was solid and trustworthy to fix the economy, even when economics experts did not find his policies and ideas beneficial. Although Harris’ defeat cannot be attributed simply to sexism, sexism is a factor worth discussing as it falls under the scope of this paper, and poses the most recent example of how leaders are treated, and commented on, differently based on their gender, as well as why a model for women leadership would be beneficial.

The comparison between the treatment of women leaders, exemplified by Marin or Harris, and men leaders like Putin or Trump, is legitimate due to its illumination of entrenched gender biases in societal perceptions of leadership, as exemplified in the literature (Eagly and Diekman, 2005; Eagly and Karau, 2002; Heilman and Okimoto, 2007; Heilman et al., 2004; Heilman et al., 1989). Both leaders—Marin and Putin--engaged in somewhat provocative public displays and both were aware that they were being photographed; yet they received vastly different reactions reflective of gendered stereotypes and expectations. Whereas negative feedback toward Marin often centered on her perceived incompetence, reactions to Putin’s photographs were framed in a positive or neutral light, highlighting traits associated with masculinity and power. This disparity underscores the existence of double standards and gender bias in at least some public perception and media portrayal, revealing systemic inequalities in leadership evaluations and societal expectations.

Because, as mentioned above, most current models of leadership take a more masculine approach, a model that acknowledges and values women’s leadership skills and traits is important for women leaders to be evaluated according to their respective strengths and successes.

### Theoretical framework of a model of successful women leadership: wisdom-intelligence-creativity-synthesized (WICS)

An effective leader is responsible for generating ideas, evaluating the advantages and disadvantages of those ideas, being able to implement these ideas, and urging others to follow those ideas. An effective leader needs to maximize the common good for all stakeholders (Sternberg, 2005a). The Wisdom-Intelligence-Creativity-Synthesized (WICS) model portrays these characteristics. It can be, and has been, applied to leadership.

Creativity refers to the skills and attitudes that are necessary for the development of novel, high-quality, and effective ideas. Intelligence refers to the skills and attitudes that are required to succeed in life, within one’s sociocultural context and one’s own understanding of success. Two aspects of intelligence that are relevant to the WICS model are practical and analytical (academic) intelligence. Academic intelligence can be defined as the memory-based and the abstract analytical skills and attitudes that form the conventional definition of intelligence. It provides the basis for evaluation and judging of information. Practical intelligence can be defined as a set of skills and attitudes needed to solve everyday problems, by using information and knowledge gained from experience (Sternberg, 2008).

Wisdom might be the most important quality for a leader to possess (Sternberg, 2005a). An individual is considered wise when ethically utilizing intelligence, creativity, and knowledge, in order to reach a common good; by balancing intrapersonal, interpersonal, and extrapersonal interests; over the short and long terms; to adapt to, shape, and select environments. Wise leaders look out for and seek a balance among their own interests, others’ interests, and the interests of the larger institutions in which they operate; wise leaders see the bigger picture. Wise leaders think long-term as well as short-term and are able to balance the needs and interests of all through the infusion of positive ethical values. Wisdom is required for good leadership because it helps a leader act on what will be beneficial for the long term, and for larger populations under the leaders’ authority.

A wise leader, as described above, seems to embody many of the virtues of a transformational leader, in terms of balancing everyone’s interests and inspiring their followers. Practical intelligence, in the relative dearth or absence of wisdom, in terms of leadership, comes closer to the transactional style of leadership because it tends to emphasize short-term, goal-oriented decision-making, effective problem-solving, and managing within existing systems–traits commonly associated with transactional approaches that reward efficiency and performance. As aforementioned (Bass et al., 1996; Burke and Collins, 2001; Goethals and Hoyt, 2016; Guillet et al., 2019), women are viewed as more likely to demonstrate transformational leadership traits, and men are more likely to demonstrate transactional leadership traits. Could this observation suggest that women might also, on average, be more wisdom-driven in regard to leadership, and men, on average, more practical-intelligence-oriented?

Practical intelligence is a core component of successful leadership (Sternberg, 2008). It includes elements like adaptation to and shaping of one’s environment, or finding a new environment within which to work, which are all skills needed to manage oneself, manage others, and manage tasks (Sternberg, 2008). Barack Obama, former President of the United States, has been characterized as a leader demonstrating exceptionally high practical intelligence (Seifi, 2014). Justin Trudeau, the Prime Minister of Canada, also was often been praised for his practical intelligence in leadership, which he demonstrated when navigating complex political landscapes with relative finesse, such as his management of the COVID-19 pandemic, climate change, and international diplomacy–though his popularity has since declined significantly. Olaf Scholz, as the Chancellor of Germany, through the various challenges he has faced, demonstrated practical intelligence by maintaining political stability and managing an economic recovery, yet faced mounting political challenges, including a coalition collapse and declining voter confidence.

Practical intelligence can also be a matter of maximizing one’s own position and gains within a role, and persuading others of one’s worth (Sternberg, 2008). Sometimes, men political leaders seem to be out to maximize their own gains; it has been suggested that Putin sometimes makes big political decisions seeking primarily or exclusively his own personal benefit (Joshi, 2014; Trenin, 2007). An example of this behavior exercised by Putin, who has already run Russia for more than 20 years, is how he managed to pass a law that allows him to stay in office until 2036, something otherwise unheard of Odynova (2021) and Roth (2021). President Xi Jinping in China also has greatly extended his tenure in a way unmatched since the days of Mao Zedong (founder of the People’s Republic of China and former chairman of the Chinese Communist Party) (Gueorguiev, 2018), maximizing his own position and gains in this way.

One might argue that, on average, women seem more wisdom-driven and less driven by personal posturing. Ardern, for example, exhibited wisdom in thought and action (Sternberg and Karami, 2021), as did Angela Merkel, who proved that sanity and wisdom, as Mayer et al. (2020) put it, can still prevail in politics–although she may have dealt with immigration and international relations in a way that was heavily criticized.

On average, women leaders may place a greater emphasis on wisdom and transformational approaches, while male leaders may more often lean on practical intelligence and transactional methods. In other words, the relative balance among the components of the WICS model may differ by gender. Within this framework, we can identify leadership characteristics that are both crucial for success and more commonly demonstrated by women. These insights are based on a broad review of literature and media accounts of both successful and unsuccessful women in leadership. The following analysis will examine current or recent women leaders, assessing the extent to which they exemplify these proposed traits.

## Methodology

For the purpose of this study, leaders from all around the world will be studied. In order to assess their success during their leadership, and to remain objective in our study, we utilized measures such as the leader’s country’s: (a) employment rates (Federal Statistical Office of Germany, 2024; MacroTrends, n.d.; Statista, 2025); (b) Freedom House ratings, derived from the official Freedom House website (Freedom House, n.d.); (c) income rates (CEIC Data, n.d.; Deutsche, 2021; Eurostat-a, n.d.; Eurostat-b, 2024; Ministry of Business, Innovation and Employment, 2024; Statista, 2023a); and (d) happiness rankings (Begley-Bloom, 2021; Countryeconomy.com-a, n.d.; Countryeconomy.com-b, n.d.; Graham, 2019; Graham, 2023; Helliwell et al., 2020; Helliwell et al., 2022; Human Progress, n.d.; Statista, 2023b). Accordingly, the women leaders referenced in this paper were selected based on quantifiable success indicators–such as employment, income, freedom, and national well-being–rather than subjective political factors or public opinion. In order to see an analytical review of how women leaders ranked in each category, please see Table 1.

**Table 1:** Measuring success in women leaders.

Leader	Gross Domestic Product (GDP)	Employment	Freedom House	Average Income	Happiness
Jacinda Ardern (New Zealand, 2017–2023)	New Zealand gdp for 2023 was $253.47B, a 2.73% increase from 2022. New Zealand gdp for 2022 was $246.73B, a 2.72% decline from 2021. New Zealand gdp for 2021 was $253.64B, a 19.25% increase from 2020. New Zealand gdp for 2020 was $212.70B, a 0.07% decline from 2019.	The unemployment rate of New Zealand experienced a decrease between 2017–2023, with a slight increase during the year 2020 when the pandemic occurred, and also a slight increase in the year 2023, following Ardern’s resignation.	In 2017, 2018, and 2019, New Zealand was rated at 98/100. In 2020, New Zealand was rated at 97/100. After that, it rated at 99/100 for 2021, 2022, 2023.	The median household income ‘skyrocketed’ during Ardern’s term. In 2016 it was at $79,900, and by 2024 it is at $122,500. The incline was steady with the exception of 2020, that had a $800 decrease from the previous year.	The World Happiness Index for New Zealand was high for the years in which Ardern was in office. Specifically, they ranked 8th in the world in 2017, and remained in that spot until 2020, followed by 9th spot in 2021, and 10th in 2022 and 2023.
Angela Merkel (Germany, 2005–2021)	In the year she took office, Germany’s GDP stood at 2.3 trillion euros ($2.6 trillion). In 2020, it was over 3.3 trillion euros.	Germany had a steady rise of employment rates from 2005 (employment rate 65.4) until 2021 (employment rate 75.6), with the only exception year 2020 where it decreased instead of increased–the year of COVID-19	Germany has been rated at a 94/100 between the years of 2018–2021, and 95/100 in the years 2016–2017. There was no available data on Germany’s Freedom House ‘Freedom in the World’ rating before 2016.	The adjusted gross disposable income of households during Merkel, rose from 21,646 euros ($23,513) in 2005, to 30,142 euros ($32,801) in 2019. The inclide was steady, with the exception of 2009 in which the income slightly decreased.	Based on the World Happiness Report, Germany has had an almost steady upward incline in terms of happiness, starting from 6.62 in 2005, and reaching 6.71 in 2021.
Sanna Marin (Finland, 2019–2023)	Finland gdp for 2023 was $300.19B, a 6.49% increase from 2022. Finland gdp for 2022 was $281.89B, a 4.92% decline from 2021. Finland gdp for 2021 was $296.47B, a 9.04% increase from 2020. Finland gdp for 2020 was $271.89B, a 1.26% increase from 2019.	Finland unemployment rate for 2023 was 7.16%, a 0.44% increase from 2022. Finland unemployment rate for 2022 was 6.72%, a 0.89% decline from 2021. Finland unemployment rate for 2021 was 7.61%, a 0.15% decline from 2020. Finland unemployment rate for 2020 was 7.76%, a 1.06% increase from 2019.	Finland has rated 100/100 in all years between 2019 and 2023. It is worth mentioning that Finland was rated at 100/100 even in prior years, however.	During Marin’s term, the household income rose from 2019 ($29,091) to 2021 ($31,845), but then had a large decrease in 2022 ($27,134).	Finland has an exceptional record of being one of the highest ranking countries in happiness, and during Marin’s time in office, the ranking remained significantly high. From 2019 until 2023, Finland was ranked 1^st^ in the world happiness ranking.
Ursula von der Leyen (European Union, 2019-present)	European Union gdp for 2022 was $16,761.50B, a 3.2% decline from 2021. European Union gdp for 2021 was $17,315.13B, a 12.57% increase from 2020. European Union gdp for 2020 was $15,381.42B, a 1.99% decline from 2019.	European Union unemployment rate for 2023 was 6.02%, a 0.13% decline from 2022. European Union unemployment rate for 2022 was 6.15%, a 0.88% decline from 2021. European Union unemployment rate for 2021 was 7.02%, a 0.02% decline from 2020. European Union unemployment rate for 2020 was 7.04%, a 0.37% increase from 2019.	*Due to the fact that the European Union is not a country in itself, and in fact consist of 27 countries, we were unable to determine a Freedom Housing rating to assess von der Leyen’s success in that domain.*	European Union household income had a slight increase from 2019 to 2020 (0.4%) due to the impact of the COVID-19 pandemic, and then increased by 2.6% in 2021, and then by 6.3% in 2022. In 2023, there was once again an increase of over 2,000 euros ($2,177), and it continues to increase in the first quarters of 2024.	European Union’s happiness index is more difficult to calculate as it comprises of many countries, however, since 2019 and until now, the majority of the 10 highest ranking countries in the world, are countries under the European Union, such as Finland, Iceland, Norway, Switzerland, and more.

## Results

This section synthesizes key findings from the review of political data and leadership traits to demonstrate how the WICS (Wisdom–Intelligence–Creativity–Synthesized) model applies to contemporary women leaders. The information compiled in Table 1 illustrates multiple metrics of national and regional success during the tenures of prominent women leaders, including Jacinda Ardern, Angela Merkel, Sanna Marin, and Ursula von der Leyen. Indicators such as GDP growth, employment trends, Freedom House ratings, average income, and national happiness indices serve as proxies for successful governance.

These economic and social metrics show a consistent pattern: under the leadership of these women, most of the countries or regions they governed saw economic stability or recovery, resilient employment levels despite global crises (e.g., the COVID-19 pandemic), and high or improving happiness scores. These outcomes reflect the application of practical intelligence in managing complex challenges and adapting to shifting environments (e.g., Marin’s COVID-19 leadership, Merkel’s long-term economic strategy, Ardern’s public communication).

In addition to measurable outcomes, Table 2 provides a qualitative analysis of leadership traits expressed by these women. Characteristics such as empathy, unity, diplomacy, humility, and resilience are strongly represented. These traits align closely with the wisdom component of the WICS model, reflecting a long-term, ethically grounded approach to leadership that considers diverse stakeholder interests. For example, Ardern’s use of inclusive language during national crises (“a team of five million”) and Merkel’s diplomatic composure during international conflicts demonstrate the integration of emotional intelligence and wise decision-making.

**Table 2:** Traits of women leaders, their definitions, and examples.

Elements—traits	Definition—explanation	Example
Three C’s: Considerate, Caring, Compassionate	Expressing consideration, compassion, and care toward one’s followers; empathizing	During Ardern’s Facebook live sessions, empathetic connection was reinforced through her communication style, by which she expressed concern about how the pandemic affected people individually
Humility	The quality of understanding oneself through awareness of personal roles, strengths, and limitations, as well as the self’s relationships with others; necessary attribute for successful leadership	Merkel hardly ever took credit for the incredible transformations her government initiated while she was in power, especially in the gender domain
Unity	Putting emphasis on uniting a nation; helps build trust	Ardern’s carefully picked phrasing–“Unite against COVID-19,” constant usage of “we,” “us,” “all New Zealanders,” and “a team of five million”
Diplomacy–emotional intelligence	The art of maintaining peaceful relationships and interactions between nations, groups, or individuals, especially during conflict and debates	Merkel has been known for her tendency always to listen, and to allow others to comment or suggest a position on a matter, before proceeding to formulate her opinion and voicing it
Resilience in the face of “misogynistic” personal attacks	Women leaders are often targeted for their looks, their clothing, their hair, or their personal lives–approaches that require resilience to deal with	Ardern being asked about her decision to dye her hair; Ardern being asked about the time of conception of her child

Taken together, the data and examples suggest that successful women leaders tend to exemplify a balance of practical intelligence (managing crises, boosting economies), wisdom (valuing collective well-being and ethical leadership), and–though less directly measured here–creativity (as seen in novel policy approaches or communications strategies). The convergence of these qualities underpins the proposed WICS-based model for women’s political leadership. While the findings presented are not the result of formal statistical analysis, they are grounded in publicly available empirical data and widely reported accounts of leadership performance. These results offer meaningful insight into patterns of success that support the proposed WICS-based model for women’s political leadership.

### Three C’s: considerate, caring, compassionate

From the WICS model, wisdom refers to striving for a common good, by the leader looking out for and balancing their own interests, others’ interests, and the interests of the larger institutions within and across which they operate. By expressing consideration and care for one’s nation, a leader demonstrates wisdom in dealing with potentially sensitive situations that affect a large group of people. There is a reason that traits such as nurturing, consideration, and caring are seen as more feminine (Bass et al., 1996), whether they are socially learned or biologically predisposed. Women tend to show more concern for others, and this tendency has also been noticed in organizational contexts: Women, as managers, demonstrate more concern for, and involvement with others, when dealing with ethical and moral considerations (Powell et al., 1984).

Jacinda Ardern served as a great example of a woman leader. At the time we started writing, she was the Prime Minister of New Zealand, a position she had held since 2017. However, during the time of initially preparing this article, Ardern announced her stepping out of office before the 2023 elections, which were to be held in October of 2023 (Diaz, 2023; Pagani, 2023). Her overall success during her time as Prime Minister has been a rather controversial topic of discussion, with some believing that she was a great Prime Minister for New Zealand, and with others arguing that although she had a strong international image, for New Zealand, she had not actually quite delivered (Pagani, 2023). It is undeniable that Ardern handled the COVID-19 pandemic with skill, leading to international admiration and popularity (Johnson and Williams, 2020; Wilson, 2020).

The COVID-19 pandemic broke out during her time in office, with the result that she had the opportunity to demonstrate compassion, care, and consideration, for the health and mental health of her nation and of the world. This caring approach could be seen in her Facebook Live sessions, which she conducted during the beginning of the pandemic. The sessions served as a medium for her to directly relate to and communicate with the people of New Zealand, as well as to answer questions and address concerns. During the sessions, empathic connection was reinforced through Ardern’s communication style, by which she expressed concern about how the pandemic affected people individually. She further answered sympathetically the questions posed to her (Johnson and Williams, 2020; Wilson, 2020). Upon the eruption of the pandemic, when information about the death toll from COVID-19 was still scarce, Ardern made a speech empathizing and feeling for the people who have been affected by COVID-19 but also promising help to international partners and friends, such as Australia. More specifically, Ardern said, “To those who have lost or are missing family and friends, we share in your grief and sorrow and we are devastated. To our international partners and friends, we will do everything we can to support you as you have supported us in times past. In particular, our family in Australia has been heavily impacted. We feel the pull of our bond acutely at this time. I say to those who have lost and grieve–you are forever linked to our nation and we will hold you close” (McLean, and Ewart, 2020). Ardern served as another woman leader case who has been described as a mother figure of a nation–the maternal protector of New Zealand who “held the citizens’ hands through the lockdown,” blending “epidemiology… with empathy,” and “empathized with citizens’ anxieties” (Johnson and Williams, 2020). Her leadership style has been described as compassionate and caring (Brown and Schockman, 2022).

Angela Merkel served as Chancellor of Germany from 2005 until 2019. As the first woman Chancellor of Germany, she managed to stay in power for 16 years. She earned titles such as “the most powerful woman in the world” (Middelhoff and Schijvenaars, 2016; Mushaben, 2017; Qvortrup, 2017). Merkel also demonstrated *compassion, empathy*, and *consideration*. During the emergence of COVID-19, Merkel openly expressed compassion for the elderly regarding how difficult isolation would be, explaining how all people are used to staying close together during hardships; she acknowledged how, this time, the opposite was required (Johnson and Williams, 2020). She has even been nicknamed as “Mutti” (Mommy). However, it is worth mentioning that Merkel has been openly criticized, especially about her last few years in office. Particularly, her biggest political mistakes are thought to be the mass migration she allowed through her open-door policy, which destabilized and upset German society, as well as her approach to Putin’s Russia post-2014 Crimea Annexation, and lastly, phasing out nuclear energy post-Fukushima in 2011 (Thurau, 2024). Therefore, it is clear that although she had many successes while in office, she made some weak choices that compromised her reliability and effectiveness, ultimately leading to her downfall after many years of leadership.

Tarja Halonen was Finland’s first woman President (Van Zoonen, 2006). Before that position, she was Finland’s first woman Minister of Foreign Affairs in 1995 (Lähteenmäki, 2017). Halonen has been described by journalists as a humane, tolerant, and kind President (Lähteenmäki, 2017). She publicly expressed her disapproval of criticizing people based on culture, language, or religion, and spoke on discrimination issues, saying how these critiques posed a disease that affected society as a whole. She worked for the Finnish Society for Sexual Equality (SETA), the Anti-Racism Commission, and the Advisory Board on Roma Affairs, projecting in this way compassion, consideration, and advocacy for minorities (Lähteenmäki, 2017). Halonen is no exception to the tendency of women leaders to be referred to as mothers of a nation; she has been praised for her nurturing and caring qualities (Van Zoonen, 2006).

Ursula von der Leyen, the first woman EU Commission President, and previously the first woman Defense Minister of Germany (Mushaben, 2022), is another distinguished woman leader of our time. During the beginning of COVID-19, she focused on *compassion* and how significant it was during those crucial and challenging times. On March 26^th^, during a speech, she emphasized how “We must look out for each other, we must pull each other through this. Because if there is one thing that is more contagious than this virus, it is love and compassion” (Donato, 2020).

A potentially relevant finding that supports the three Cs in terms of women leadership during COVID-19 is that it has been found that when women are involved in wise prevention and crisis response, it typically results in better outcomes and lowers risk; the women play a significant role in the community’s everyday life and have greater insight into the needs of the most vulnerable (Haycox, 2019, as cited in Subert, 2020), demonstrating in this way a compassion for and understanding of the community and its needs. Even though women leaders occupy approximately only 7% of world leaders, it has been suggested that women leaders have done on average a more successful job than men with handling the emergence of COVID-19 (Craig, 2021; Panayiotou, 2020). Nations led by women had significantly lower death rates. Merkel also seems to have stood by the claim that women may have better outcomes when dealing with crises, as in her response to the company IBM not, at the time, having given top-level managerial jobs to women. She said, “Perhaps IBM must first go through a real crisis before a woman is allowed to take over the company leadership” (Thompson and Lennartz, 2006). It should be noted that since then, IBM had a woman CEO for approximately 8 years–Ginni Rometty (Murray and Fordyce, 2023).

### Humility

Humility, which can be defined as the personal quality of understanding oneself through awareness of personal roles, strengths, and limitations, as well as the self’s relationships with others (Nielsen et al., 2010), has been suggested by leadership experts and management consultants as a necessary attribute for successful leadership (Collins, 2001; Morris et al., 2005; Owens et al., 2013). Women have been rated by others to be humbler, on average, than are men (Michalec et al., 2021; Peters et al., 2011). Additionally, the concept of humility is included in this model due to its positive correlation with other significant leadership traits. A study found that humility was an accurate predictor of one’s ability to be cooperative and collaborative, as well as of people’s own individual performance (Owens et al., 2013). Expressed humility was also found to reflect openness to feedback and appreciation of others’ strengths, as well as to improvement in performance (Owens et al., 2013). However, one could argue that many men leaders show a distinct lack of humility. Donald Trump, Vladimir Putin, or current President of Turkey and former Prime Minister of Turkey Recep Tayyip Erdoğan, rarely, if ever, have admitted to making mistakes. They also have what have been termed autocratic styles of leadership (Pinto, 2024).

Angela Merkel, throughout her time in office, demonstrated humility, and even expressed it as her favorite virtue (“Demut”)–she hardly ever took credit for the incredible transformations the German government underwent while she was in power, especially in the gender domain (Mushaben, 2022). When asked whether she would describe herself as a feminist, she replied by saying, “I do not want to adorn myself with a title that I do not have … Alice Schwarzer, and others, they have fought really hard battles, then I just come along and sit on top of their successes and say ‘I am a feminist’ …” (Mushaben, 2022). Additionally, as Struve (2015) suggested, Merkel’s humility, which has not been common among political leaders in Germany, served as a means for her followers to be able to relate to her more, and to admire her for her humility and for not trying to elevate herself above them.

Ardern showed that she was a *humble* Prime Minister. She said, “If you do not humble yourself, everyone else will do it for you […]” (Chapman, 2020). Her humility has also been compared to Obama’s, as he was also a leader who stood by people as a friend, confidant, and mourner (Robins, 2020). Ardern approached the pandemic through willingness to consult and listen to health experts, and upon receiving praise for her great crisis leadership, she humbly denied such claims and admitted that she had just shown humanity (Panayiotou, 2020; Shams, 2020). She said, “You need to remove some of the politics sometimes and just think about humanity. That’s all” (Shams, 2020).

Sanna Marin, at the time, the world’s youngest Prime Minister (at the age of 34) (Palonen, 2020), was also faced with the emergence of COVID-19. Marin, especially in the beginning of the pandemic, engaged in frequent statements expressing uncertainty regarding scientific knowledge about the pandemic. Even though this approach has been described as rare in the political scene of Finland; one could argue that it portrayed an unreliable and insecure political figure in contrast to other, overconfident politicians, as Parviainen et al. (2021) suggested, this could be a case of “epistemic humility.” This approach to the worldwide health crisis emphasizes the significance of relying on experts’ knowledge of subject matter, and portrays Marin as relatable and humble.

Halonen also demonstrated *humility*; she openly chose to regard herself as a servant of the people, a title which made her seem humble and down-to-earth. Halonen claimed during debates regarding Finland’s future that “Finland does not go bragging of its past in Europe.” She has been fairly described as having a down-to-earth demeanor (Lähteenmäki, 2017).

### Striving for unity

Another characteristic that we may be able to observe among great women leaders is an emphasis on the concept of unity (Mayer and Niekerk, 2020). Many of the women leaders seem to possess a need to unite their nation. De la Rey (2005) discussed how Stanford et al. (1995) showed that women leaders tend to be characterized as participative leaders and to encourage a high degree of employee involvement within their groups. Eagly and Johnson (1990) also argued that women tend to adopt more democratic or participative styles, whereas men are slightly more autocratic or directive.

Jacinda Ardern’s government, faced with the emergence of COVID-19, placed a lot of emphasis on the concept of *unity*: “Unite against COVID-19.” This can even be seen in speech details, such as the constant usage of “we,” “us,” “all New Zealanders,” and “a team of five million”–all phrases that highlight how significant it is to be thinking about the whole nation as a united team, fighting together (Craig, 2021; Wilson, 2020). The women leaders’ emphasis on the significance of unity has helped the people build trust. This sense of inclusiveness was a notable feature of Ardern’s leadership style (Simpson et al., 2022). Her leadership style also has been described as team-focused (Brown and Schockman, 2022). In contrast, there have been many men leaders who have taken a “You are with us, or you are against us” attitude, as in all the current dictatorships, which are almost uniformly led by men.

A prominent characteristic of Angela Merkel was her emphasis on the pattern of *unity*. During her years in office, Merkel focused a lot on bringing the German nation together, and she strived to be known as a “chancellor of all Germans” (Spiegel, 2005, as cited in Mayer and Niekerk, 2020). During COVID-19, Merkel used a televised national address to stress the importance of “the need to work together” among all Germans (Beaubien, 2020, as cited in Blake-Beard et al., 2020).

Von der Leyen also has paid a lot of attention to the concept of *unity*–she has aimed to create a more collaborative and united environment for the European Union, while simultaneously respecting all the different nation-states comprising it (Donato, 2020). Von der Leyen has openly noted that the European Commission should become “more united” in its nature (Zwolski, 2020).

In the United States, which is dominated by men leaders, almost all policy matters have come to be seen by citizens through a partisan lens, even when it is to the country’s and people’s detriment, such as for COVID-19 and other matters of public health (Grossman et al., 2020). There are some women leaders in the U. S. who have taken a highly partisan stance, such as Marjorie Taylor Greene and Kari Lake, but they are generally imitative, not altogether successfully, of the macho style (and level of competence) of Trump (Godfrey, 2022).

### Diplomacy–emotional intelligence

Whether it is a biologically or a socioculturally determined characteristic, or both, multiple studies with various samples, from managers to adolescents in Chandigarh, India, have demonstrated that women tend to have higher emotional intelligence test scores, on average, than do men (Katyal and Awasthi, 2005; Mandell and Pherwani, 2003; Petrides and Furnham, 2000). Emotional intelligence may engender a whole range of qualities to people who possess it–being diplomatic serves as an excellent example of a skill that requires emotional intelligence (Kumar et al., 2012; Polychroniou, 2009). It is a great asset for a leader to possess (Katz et al., 2011; Prestia, 2017). Wisdom is associated with emotional intelligence. Emotions provide important information and can help guide decision-making and behaviors in complex situations. Many situations that require wisdom evoke emotions in the people involved and hence require knowledge and skills to manage and regulate these emotions (Kunzmann and Glück, 2019). Additionally, even though the WICS model of leadership focuses on practical and analytical intelligence, it also incorporates elements of emotionally intelligent leadership through practical intelligence (Sternberg, 2005b).

Merkel often was praised for her diplomatic skills–in fact, she has been described as having the ability to “reduce the tension in a meeting room, not to ratchet it up” (Yoder, 2011). Merkel has been known for her tendency always to listen, and to allow others to comment or suggest a position on a matter, before proceeding to formulate her opinion and voicing it (Mayer and Niekerk, 2020). She often engaged in “strategic maneuvering,” by allowing others such as von der Leyen to take the lead, due to recognition that each policy decision requires knowledge from a specific context (Mushaben, 2022).

### Resilience in the face of “misogynistic” personal attacks

Another factor that unites women leaders is a resilience that they share when they are faced with attacks regarding their personal appearance, their clothing, their hair, and their personal relationships (Mavin et al., 2010; Sakki and Martikainen, 2021; Saluja and Thilaka, 2021). Women in the public sphere, especially in positions of political leadership, receive comments on all kinds of personal things that men rarely receive. This type of commentary goes beyond just gossiping about one’s sense of style–it is a gendered construction in mass media that discourages women’s accessibility and credibility in positions of power, as their contributions go unnoticed or trivialized; instead, their physical appearance becomes the primary topic of discussion (Mavin et al., 2010). This type of commentary could be described as misogynistic--an attempt to put into words targeted critiques that are almost exclusively women-directed. A high level of resilience is needed to deal with and effectively combat these types of attacks.

As aforementioned, leadership involves both skills and attitudes. Based on the WICS model, creativity entails some elements that generate a creative leader (Sternberg, 2005b). As Sternberg (2005b) argued, an element of a creative attitude toward leadership is willingness to surmount obstacles and to show courage when opposition becomes nasty and punitive. Even though it is not rare for a leader to encounter obstacles (e.g., misogynistic personal attacks), the gifted leader embodies a great amount of resilience in order to proceed and keep working toward their goals and the common good.

An example portraying this type of personal attack that women leaders often receive is the aforementioned case of Marin, who received backlash for a photoshoot (Sakki and Martikainen, 2021). Another example of such commentary is the incident at Auckland’s Government House in New Zealand, in which Prime Ministers Ardern and Marin were asked by a journalist whether they were meeting due to their similarity in age (Corlett, 2022). Ardern called out the journalist and wondered out loud whether anyone had ever asked Obama and Former Prime Minister of New Zealand John Key if they met due to their similar age. Marin responded with, “We’re meeting because we are prime ministers.” Ardern also was asked, when pregnant, when her baby was due, in an attempt to figure out the conception date, as the reporter openly admitted; she was also asked by reporters about potentially getting married, or questioned about dying her hair color–questions that have nothing to do with her political role and duties and that men would be unlikely to be asked. Donald Trump has a highly unusual orange hair color, but it is not routinely, or perhaps, ever asked how it came to be.

Additionally, an impressive amount of press space was devoted Clinton’s physical appearance, some titled “Hillary Clinton is not a lesbian–but she dresses like one” (Urquhart, 2016), “From funky florals to over-the-top ruffles: FEMAIL reveals Hillary Clinton’s 20 WORST fashion faux pas from the past 50 years” (Tempesta, 2015), or “Hillary Clinton wore a $12,495 Armani jacket during a speech about inequality,” (Whitten, 2016), to name just a few insults about her personal appearance.

### Potential limitations deriving from those characteristics

As it is important to provide some balance, this article also aims to recognize some potential limitations that may derive from the aforementioned traits. Almost all characteristics have multiple sides–they work sometimes but not others. For example, Ardern started off well; then citizens started to feel like she was short on delivery of what she had promised. Although she seemed to possess all characteristics mentioned above, still, at the end of the day, as of January 2023, she decided to file her resignation (Diaz, 2023). She announced that she was stepping down because she acknowledged that she did not have enough “in the tank” to do her job justice. The *New York Times’* article titled “Jacinda Ardern, the Star Who Didn’t Quite Deliver,” examined how even though she was an empathetic and promising leader who was admired around the world, many New Zealanders came to feel that her actions did not match her promises (Pagani, 2023). The article argued that Ardern dealt with COVID-19 successfully and managed to be well-liked internationally; but when it came to substantial changes within New Zealand, the progress was not as significant as one would have hoped for, in matters of children living in material hardship, average wages, and new infrastructures, among other aspects.

### The limits and contexts of leadership success

Even the most competent and celebrated leaders are susceptible to making mistakes, and over time, these missteps can lead to a decline in their influence or effectiveness. This is not an exception but a common pattern observed across leadership trajectories. The demands of leadership, evolving expectations, and accumulated pressures often result in decision-making errors, misjudgments, or an eventual mismatch between the leader’s style and changing contextual needs.

Leadership effectiveness is not a fixed trait but is instead shaped by the dynamic interaction among the individual, the specific task at hand, the context or situation, and the audience involved. A behavior or trait that proves effective in one scenario may fail in another due to shifts in any of these variables. This person × task × situation × audience framework provides a valuable lens for understanding why leadership strategies must be flexible and context-sensitive. As Sternberg et al. (2023) argue, intelligence, creativity, and wisdom are not static capacities but are situated in these interactions. Therefore, leadership that thrives in one set of conditions may falter when the conditions evolve or when applied rigidly across differing contexts (Sternberg et al., 2023). This pattern holds true across genders–both men and women leaders can find that the very traits that once brought success may later contribute to their challenges or downfall, depending on the evolving context.

### Implications for research and practice

The WICS-based model of women’s leadership has significant implications for both research and practice, particularly within the realm of political leadership. First, future research should more deeply explore how wisdom, intelligence (especially emotional intelligence), and creativity manifest in women political leaders across different cultural and geopolitical contexts. Comparative analyses between women and men in high political office can help identify whether the observed patterns in leadership styles (e.g., transformational vs. transactional) hold consistently or vary with context, ideology, or policy domain.

Practically, this model suggests that political institutions and leadership development programs should broaden their evaluative criteria for leadership potential. Rather than emphasizing task orientation or practical intelligence–traits often aligned with traditional masculine leadership norms–there should be greater recognition of wisdom-based leadership, emotional intelligence, and creative problem-solving as critical indicators of effectiveness. Mentorship programs for women entering politics can also draw on the WICS framework to cultivate a more holistic set of leadership capacities.

Finally, policymakers and the public alike may benefit from frameworks like WICS that challenge gendered assumptions about leadership by emphasizing cognitive and ethical dimensions over purely performative ones. By doing so, the model promotes more inclusive understandings of political success and opens the door for a wider range of leadership styles to be legitimized in the public arena.

## Discussion and conclusion

This article and the theoretical project underlying it does not imply that women leaders are the only successful leaders, or that they are more successful in leadership roles than men. Successful women leaders were utilized in this paper as examples of women leaders exhibiting the characteristics proposed in the model. However, there are women leaders who have not been considered successful during their leadership, and of course there are men who have been considered very successful during their leadership. A recent example of a woman who is not considered to have been well-regarded due to her leadership choices was Bangladesh’s former prime minister Sheikh Hasina, whose regime was characterized as fascist and authoritarian, and who also fled the country following a series of deadly and violent anti-government protests (Vock and Ethirajan, 2024). Hasina failed to maintain stability in the country and to address public grievances, leading to widespread disconnect and violence (Vock and Ethirajan, 2024). It was also an authoritarian regime, unlike most of the regimes led by women.

Another example of a controversial woman leader is Argentina’s former president, former first lady, and former vice-president Cristina Fernández de Kirchner. She has been described as “the personification of evil, a Machiavellian devil whose only purpose in life is to perpetuate herself in power for personal gain” (Fontevecchia, 2022). Kirchner has been convicted of a crime while in office, as in December 2022 she was found guilty of awarding public contracts worth $1bn to close friends (Profile: Cristina Fernández de Kirchner, 2022), and has been sentenced for corruption and permanently banned from holding public office (Rascoe, 2025). These examples demonstrate that leadership success and public perception are influenced by a variety of factors beyond gender, highlighting that both men and women can experience varying levels of effectiveness and popularity in leadership roles.

Undoubtfully, there are a number of exceptional women leaders, current or past, whose work in the political sphere is admired. Scholars like Eagly and Carli (2003, 2007), Eagly and Karau (2002), Eagly and Diekman (2005), Heilman et al. (1989, 2004), and Wang et al. (2013), among others, have thoroughly discussed aspects of women leadership, such as gender stereotypes, transformational vs. transactional leadership style, limitations, and prominent women leadership characteristics, adding to the ever-evolving research of the field. There is no doubt that women in top leadership positions have had to face some discrimination and doubts, stemming from their gender, and the stereotypes that follow it. However, things are changing, research is evolving, and stereotypes are breaking. The WICS model of leadership (Sternberg, 2005a) seems to conceptualize satisfactorily women leadership, and poses a well-defined theoretical framework for our model. The three C’s (considerate, caring, compassionate), humility, striving for unity, diplomacy (emotional intelligence), and resilience in the face of misogynistic personal attacks, are traits and behaviors drawn from the WICS model of leadership, and often have been expressed by women leaders as analyzed above.

In conclusion, this essay has delved into the evolving landscape of leadership, recognizing the profound shift in gender dynamics within this sphere, and discussing prevalent topics within the realm of women leadership. By employing the WICS model, this essay elucidates the significance of qualities such as compassion, humility, unity, diplomacy, and resilience in successful women leaders. As exemplified by figures like Marin, Halonen, and von der Leyen, among others, women have displayed exceptional prowess in leadership, challenging preconceived notions and carving their path towards progress. Their accomplishments underscore the importance of embracing diverse leadership styles, and of a society opening up to, and praising more, stereotypically female characteristics that seem to be necessary in leadership, while not undermining more stereotypically male characteristics that seem to be necessary in leadership.
